# Richness of Lichen Species, Especially of Threatened Ones, Is Promoted by Management Methods Furthering Stand Continuity

**DOI:** 10.1371/journal.pone.0055461

**Published:** 2013-01-29

**Authors:** Steffen Boch, Daniel Prati, Dominik Hessenmöller, Ernst-Detlef Schulze, Markus Fischer

**Affiliations:** 1 Institute of Plant Sciences and Botanical Garden; University of Bern, Bern, Switzerland; 2 Oeschger Center, University of Bern, Bern, Switzerland; 3 Max-Planck-Institute for Biogeochemistry, Jena, Germany; Institute of Botany, Czech Academy of Sciences, Czech Republic

## Abstract

Lichens are a key component of forest biodiversity. However, a comprehensive study analyzing lichen species richness in relation to several management types, extending over different regions and forest stages and including information on site conditions is missing for temperate European forests. In three German regions (Schwäbische Alb, Hainich-Dün, Schorfheide-Chorin), the so-called Biodiversity Exploratories, we studied lichen species richness in 631 forest plots of 400 m^2^ comprising different management types (unmanaged, selection cutting, deciduous and coniferous age-class forests resulting from clear cutting or shelterwood logging), various stand ages, and site conditions, typical for large parts of temperate Europe. We analyzed how lichen species richness responds to management and habitat variables (standing biomass, cover of deadwood, cover of rocks). We found strong regional differences with highest lichen species richness in the Schwäbische Alb, probably driven by regional differences in former air pollution, and in precipitation and habitat variables. Overall, unmanaged forests harbored 22% more threatened lichen species than managed age-class forests. In general, total, corticolous, and threatened lichen species richness did not differ among management types of deciduous forests. However, in the Schwäbische-Alb region, deciduous forests had 61% more lichen species than coniferous forests and they had 279% more threatened and 76% more corticolous lichen species. Old deciduous age classes were richer in corticolous lichen species than young ones, while old coniferous age-classes were poorer than young ones. Overall, our findings highlight the importance of stand continuity for conservation. To increase total and threatened lichen species richness we suggest (1) conserving unmanaged forests, (2) promoting silvicultural methods assuring stand continuity, (3) conserving old trees in managed forests, (4) promoting stands of native deciduous tree species instead of coniferous plantations, and (5) increasing the amount of deadwood in forests.

## Introduction

The main reasons for the ongoing decline of lichen species richness are habitat degradation caused by human interference such as disturbances by management activities, and air pollution. Together these factors have resulted in large numbers of threatened lichen species [1]–[3]. Because of their sensitivity to land-use and habitat changes lichens are very important environmental indicators [2]. In particular, rare and threatened species should be considered in conservation-oriented forest-management plans [4], [5]. Between the 1950’s and 1980’s, air pollution was high in certain parts of Europe (e.g. [6]). In particular the extent of sulfur dioxide (SO_2_) deposition, which reduced bark pH and regional lichen species pools [7]–[9], differed strongly among Central European regions. Hence, studying different regions is required to reach general conclusions about forest-management effects on lichen diversity.

Temperate European forests have long management histories, and forests without human influence are restricted to remote or inaccessible areas [10]. Hence, European forests are fragmented with large areas dominated by economically profitable age-class forests. These contain stands with even-aged tree structure, which had either resulted from clear cuts or from shelterwood logging. In the second case all trees of a stand are removed in a series of two cuts: first, approximately 60% of the tree cover is harvested, leaving scattered shelter trees for seedlings and secondly, after establishment of a closed stand cover of young trees, the remaining shelter trees are also removed. Such even-aged forest stands are often managed as plantations of fast growing conifers [11]. As both methods replace the whole stand by a new tree layer in a relatively short time period, stands are non-continuous in both cases. In recent years, silvicultural methods were promoted which attempt to mimic the natural-forest cycles typical for a particular vegetation zone. These methods focus on reproducing natural gap dynamics and regeneration and on promoting original vegetation, such as mixed forests dominated by deciduous tree species [10], [12]. In comparison to conventional clear cuts and shelterwood logging, which result in age-class forests, silvicultural methods such as selection cuttings assure continuous tree cover by harvesting single trees or small groups of trees, thus promoting uneven-aged stands. Selection cuttings should maintain and enhance biodiversity in forests whilst producing timber in an economically efficient way [12].

In Europe the area of protected forest reserves without management has recently been increased, mainly to conserve vulnerable and rare forest ecosystems, and to establish a reserve network [10]. However, because nearly all of these forests were formerly more or less intensively managed. Central European unmanaged forests are not comparable with natural forests in North America, Siberia or some parts of Eastern and Northern Europe which have been largely untouched by man for centuries [12]. In temperate European forests the efficacy of forest protection and of different silvicultural systems for maintaining lichen diversity is still poorly investigated, calling for a comprehensive analysis [13].

Lichens, symbiotic associations between mycobiontic and photobiontic partners, occur on a wide range of substrates in most terrestrial ecosystems of the world, including the bark of trees (corticolous lichens), soil (terricolous lichens), rocks (saxicolous lichens) and deadwood (lignicolous lichens). Nevertheless, many particular lichen species are restricted to a narrow ecological niche with specific requirements concerning substrate (e.g. bark, deadwood, rocks, soil), pH value, and nutrient status. Thus, particular habitats, and even successional stages within habitats, harbor distinctive lichen communities with successional variation in their lichen composition [2].

Case studies in North America and Europe showed higher corticolous and threatened lichen species richness in unmanaged than in managed forests [13]–[15]. In addition, it was suggested that silvicultural systems assuring stand continuity of forests, such as selection cutting or prolonged rotation periods, might maintain and increase lichen species richness and should therefore be favored over conventional forestry methods including clear cuts [16], [17].

Unfortunately, to date there have been no comprehensive, comparative studies from temperate European forests on the response of lichen species richness to management. No studies have included different regions, management types and developmental stages, along with detailed information on site conditions [13]. Furthermore, rather than addressing all lichen species in defined areas of differently managed forests, only corticolous species on individual trees were usually recorded [18], [19]. In addition, studies on the effects of stand age or stand characteristics on lichen species richness are rare outside Fennoscandia, where case studies have been carried out (e.g. [20]–[23]).

We present a comparative study analyzing the response of the species richness of all lichens, of lichens separated by substrate (corticolous, lignicolous, saxicolous), and of threatened lichens to management and habitat variables (standing biomass, cover of deadwood, cover of rocks). This is the most extensive lichen dataset from Central Europe to date.

Our main questions are:

(1) How does lichen species richness respond to forest management?

(2) How does lichen species richness respond to habitat variables?

## Materials and Methods

### Study system

This study was conducted as part of the Biodiversity Exploratories project (www.biodiversity-exploratories.de) in three German regions: (1) the UNESCO Biosphere area Schwäbische Alb (Swabian Jura), situated in the low mountain ranges of South-western Germany (2) the National Park Hainich and its surrounding areas, situated in the hilly lands of Central Germany, and (3) the UNESCO Biosphere Reserve Schorfheide-Chorin, situated in the young glacial lowlands of North-eastern Germany. The three study regions differ in climate, geology, and topographical situations and harbor land uses as well as species pools typical for large parts of temperate Europe ([24]; Table 1). Past mean annual SO_2_ depositions had been low in the Schwäbische Alb, high in Schorfheide-Chorin, and very high in Hainich-Dün ([25]; Table 1).

**Table 1:** Main geographic and habitat characteristics of the three Biodiversity Exploratories.

	Schwäbische Alb	Hainich-Dün	Schorfheide-Chorin
Location	SW Germany	Central Germany	NE Germany
Size	∼422 km^2^	∼1300 km^2^	∼1300 km^2^
Geology	Calcareous bedrock	Calcareous bedrock	Young glacial landscape
Altitude a.s.l.	460–860 m	285–550 m	3–140 m
Annual mean temperature	6.0–7.0 °C	6.5–8.0 °C	8.0–8.5 °C
Annual mean precipitation	700–1000 mm	500–800 mm	500–600 mm
SO_2_ deposition			
until 1985	25–<50 µg/m^3^	>150 µg/m^3^	25–<50 µg/m^3^
1985 to 1990	<25 µg/m^3^	>150 µg/m^3^	50–<75 µg/m^3^
1990 to 1995	<25 µg/m^3^	25–<50 µg/m^3^	50–<75 µg/m^3^
since 1995	<25 µg/m^3^	<25 µg/m^3^	<25 µg/m^3^
Number of plots	152	172	307
Standing biomass [m^3^/ha]			
mean (SD)	336.7 (187.8)	408.6 (188.9)	444.8 (187.0)
range	1.2–1017.4	3.9–881.5	18.3–1001.5
Cover deadwood [%]			
mean (SD)	3.6 (3.0)	3.1 (2.8)	3.8 (3.6)
range	0.5–20.0	0.5–15.0	0.5–25.0
Cover rocks [%]			
mean (SD)	1.0 (1.6)	0.2 (0.5)	0.3 (0.6)
range	0.0–11.0	0.0–4.0	0.0–5.5

### Plot selection

Each region, of at least 20 km by 30 km, contains more than 500 forest plots selected from the intersection points of a 100 m×100 m grid, after discarding plots fully or partially overlapping with settlements, grasslands, agricultural fields, water bodies and plots intersected by roads [24]. From these plots, we randomly selected 631 plots for this study, which cover all management types in each region: 152 in the Schwäbische Alb, 172 in Hainich-Dün, and 307 in Schorfheide-Chorin. Thus, we consider our plot sample as unbiased with regard to studying differences in forest management.

### Management data

To assess the management system and stand characteristics of forests, a forest inventory had been conducted on a circular area of 500 m^2^ (radius 12.62 m) in each plot. Unmanaged forests were mature, deciduous forests dominated mainly by European beech *(Fagus sylvatica)*. Age-class forests were dominated by European beech, Norway spruce *(Picea abies)* or Scots pine *(Pinus sylvestris)* and had different developmental stages of even-aged structure due to harvests at 80- to 120-year intervals. Selection forests were uneven-aged deciduous stands dominated by European beech, in which single or small groups of trees were harvested selectively. As stand characteristics, we counted the number of trees (>7 cm diameter at breast height; DBH), measured their DBH, and their height using an ultrasonic tree height meter (Vertex III Forester, Haglöf, Langsele, Sweden). We then calculated standing biomass (m^3^/ha) using height and DBH of each occurring tree accounting for tree species specific trunk shapes (for details see [26]). Standing biomass can be used as a combined indicator for both tree densities (negative relation with standing biomass; [27]) and stand age (positive relation with standing biomass). We additionally recorded the percentage ground covered by rocks and deadwood respectively as indicators of substrate quantity. Furthermore, we recorded the occurrence of logging trails. Spacing of logging trails turned out to be about 20 m in coniferous and about 40 m in deciduous forests.

### Vegetation data

During 2007 and 2008 the first author recorded lichens in all 631 plots. In each case lichens were recorded on 20 m×20 m (in the center of each plot and concentric with the forest inventory circle). All lichen species per plot were identified and lichens were also recorded separately for each of the four substrate categories: bark (corticolous species, up to 2.5 m height on tree trunks and branches of shrubs), rocks (saxicolous species), deadwood (lignicolous species), and soil (terricolous species), resulting in total and substrate-specific richness values. Further, we obtained the number of lichen species classified as critically endangered to vulnerable in the red list of threatened lichens of Germany [1]. As we recorded very few terricolous lichen species we considered them when analyzing total lichen species richness but, in contrast to the other groups, did not analyze them separately.

As an additional measurement of habitat diversity for corticolous lichen species, we recorded the number of tree species (>5 m height) per plot, estimated their percentage cover and summed these cover estimates (cumulative tree cover). Across age-class forests we used the proportion of coniferous tree cover, to separate coniferous (≥70%; *N*  =  115) and deciduous age-class forests (including mixed and pure deciduous stands; *N*  =  379).

### Statistical analysis

We analysed the response of lichen species richness to management and habitat variables. Response variables were the species richness of all lichens and the species richness of the separate lichen groups (corticulous, saxiculous and ligniculous). Explanatory variables were management type, total cover of rocks, total cover of deadwood, standing biomass, age of the oldest tree per plot (max. DBH/plot), the number of tree species, and cumulative tree cover. As we were analyzing count data we used GLM models with quasi-Poisson errors to correct for overdispersion. We used subsets of the data for three separate analyses to compare effects of different management types: (1) unmanaged vs. age-class forests (all regions), (2) unmanaged vs. deciduous age-class vs. selection forests (without Schorfheide-Chorin, where no selection forests were available in the dataset), and (3) coniferous age-class vs. deciduous age-class forests (all regions). Furthermore, we included interactions with region, management type and standing biomass. Sequential F-tests were used to test the significance of deviance changes associated factors added progressively to the model (the sequence is shown in Tables 2, 3, 4). Covariables (cover of rocks and cover of deadwood) were fitted before management, meaning that the management effect is corrected for these variables and effects of management on lichens are not due to differences in the amount of deadwood or rocks between management types. As the number of tree species per plot and the occurrence of logging trails had no effect on lichen species richness in our study, we removed these variables from the analyses. We also excluded maximum DBH and cumulative tree cover because these were correlated with standing biomass (for both variables: *r*  =  0.623, *p* < 0.0001). Data were analyzed using *R*, Version 2.13.1 [28].

**Table 2:** GLM results for differences in lichen species richness between 86 unmanaged and 494 age-class forests.

		Species richness									
		All lichens		Threatened lichens		Corticolous lichens		Saxicolous lichens		Lignicolous lichens	
Source of variation	df	*F*	*p*	*F*	*p*	*F*	*p*	*F*	*p*	*F*	*p*
Region	2	435.42	**<0.001**	275.47	**<0.001**	301.85	**<0.001**	57.135	**<0.001**	41.538	**<0.001**
Rock cover	1	23.75	**<0.001**	16.60	**<0.001**	––	––	138.140	**<0.001**	––	––
Deadwood cover	1	1.68	0.196	16.87	**<0.001**	––	––	––	––	11.704	**0.001**
*Management*											
Management (unmanaged vs. age class)	1	0.32	0.572	20.61	**<0.001**	0.424	0.515	23.047	**<0.001**	5.460	**0.020**
Standing biomass	1	3.43	0.065	7.11	**0.008**	14.970	**<0.001**	0.005	0.944	25.587	**<0.001**
Management × standing biomass	1	0.06	0.812	0.25	0.615	0.160	0.690	0.697	0.404	2.265	0.133
*Regional interactions*											
Region × rock cover	2	0.18	0.839	3.76	**0.024**	––	––	13.711	**<0.001**	––	––
Region × deadwood cover	2	13.00	**<0.001**	2.85	0.059	––	––	––	––	0.036	0.965
Region × management	2	1.24	0.290	4.38	**0.013**	2.332	0.098	10.800	**<0.001**	0.412	0.663
Region × standing biomass	2	1.25	0.287	5.07	**0.007**	1.710	0.182	2.330	0.098	4.003	**0.019**
Residual Deviance	≥564	1072.40		814.86		1045.13		605.33		840.47	

Significant differences are indicated by bold *p* values.

**Table 3:** GLM results for differences in lichen species richness among 516 deciduous forests (86 unmanaged vs. 379 age-class vs. 51 selection forests).

		Species richness										
		All lichens			Threatened lichens		Corticolous lichens		Saxicolous lichens		Lignicolous lichens	
Source of variation	df	*F*		*p*	*F*	*p*	*F*	*p*	*F*	*p*	*F*	*p*
Region	1	717.40	**<0.001**		479.84	**<0.001**	495.94	**<0.001**	133.81	**<0.001**	26.51	**<0.001**
Rock cover	1	4.85	**0.028**		5.70	**0.018**	––	––	83.47	**<0.001**	––	––
Deadwood cover	1	2.54	0.112		0.65	0.423	––	––	––	––	6.33	**0.012**
*Management*												
Management (Unmanaged vs. age class vs. selection)	2	0.31	0.731		7.15	**0.001**	0.34	0.714	2.14	0.119	0.23	0.794
Standing biomass	1	10.19	**0.002**		19.74	**<0.001**	22.12	**<0.001**	0.14	0.712	22.72	**<0.001**
Management × standing biomass	2	3.81	**0.023**		1.40	0.248	1.66	0.193	3.00	0.052	1.34	0.263
*Regional interactions*												
Region × rock cover	1	0.03	0.859		4.39	**0.037**	––	––	14.26	**<0.001**	––	––
Region × deadwood cover	1	10.20	**0.002**		1.33	0.250	––	––	––	––	0.41	0.521
Region × management	2	4.45	**0.013**		5.46	**0.005**	7.97	**<0.001**	4.68	**0.010**	0.10	0.908
Region × standing biomass	1	0.02	0.901		3.83	0.051	0.50	0.481	0.14	0.709	0.00	0.983
Residual Deviance	≥272	540.05			370.10		493.88		359.15		332.63	

Significant differences are indicated by bold *p* values.

**Table 4:** GLM results for differences in lichen species richness between 379 deciduous and 115 coniferous age-class forests.

		Species richness									
		All lichens		Threatened lichens		Corticolous lichens		Saxicolous lichens		Lignicolous lichens	
Source of variation	df	*F*	*p*	*F*	*p*	*F*	*p*	*F*	*p*	*F*	*p*
Region	2	392.86	**<0.001**	284.51	**<0.001**	277.88	**<0.001**	47.73	**<0.001**	35.86	**<0.001**
Rock cover	1	27.88	**<0.001**	19.58	**<0.001**	––	––	83.02	**<0.001**	––	––
Deadwood cover	1	1.44	0.231	14.39	**<0.001**	––	––	––	––	7.28	**0.007**
*Management*											
Management (deciduous vs. coniferous age class)	1	16.20	**<0.001**	63.94	**<0.001**	39.13	**<0.001**	5.28	**0.022**	26.00	**<0.001**
Standing biomass	1	4.34	**0.038**	14.15	**<0.001**	16.81	**<0.001**	0.29	0.592	25.82	**<0.001**
Management × standing biomass	1	11.62	**0.001**	20.67	**<0.001**	10.12	**0.002**	3.11	0.078	0.68	0.411
*Regional interactions*											
Region × rock cover	2	0.35	0.704	3.22	**0.041**	––	––	6.21	**0.002**	––	––
Region × deadwood cover	2	9.30	**<0.001**	2.26	0.105	––	––	––	––	0.20	0.817
Region × management	2	6.07	**0.002**	0.92	0.401	8.34	**<0.001**	3.04	**0.049**	1.10	0.334
Region × standing biomass	2	0.20	0.821	2.06	0.129	0.05	0.953	1.03	0.357	7.00	**0.001**
Residual Deviance	≥478	896.93		582.89		846.33		435.47		688.15	

Significant differences are indicated by bold *p* values.

## Results

### Overall and regional lichen species richness

We recorded 202 lichen species, including 73 which are threatened in Germany. Of these 202 species, 124 were corticolous, 84 lignicolous, 59 saxicolous and 18 terricolous. Across all 631 plots the species richness of corticolous lichens was positively correlated with the species richness of lignicolous and saxicolous lichens (lignicolous versus corticolous, *r*  =  0.0838, *p*  =  0.0354; corticolous versus saxicolous, *r*  =  0.3091, *p* < 0.0001).

In the Schwäbische Alb region we recorded 177 species, in Hainich-Dün 59, and in Schorfheide-Chorin 70. Thirty-three of the recorded species were found in all three regions, 52 species were shared by the Schwäbische Alb and Hainich-Dün regions, 47 by the Schwäbische Alb and Schorfheide-Chorin regions, and 36 by the Hainich-Dün and Schorfheide-Chorin regions. One-hundred and eleven species were recorded exclusively in the Schwäbische Alb region, 7 in Hainich-Dün, and 23 in Schorfheide-Chorin.

**Figure 1:**
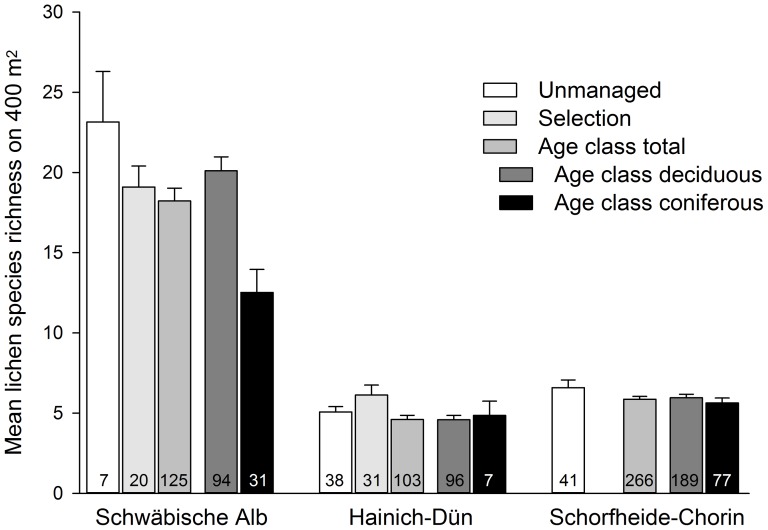
Mean lichen species richness (+SE) per plot for each of the forest management types in the three study regions.

**Figure 2:**
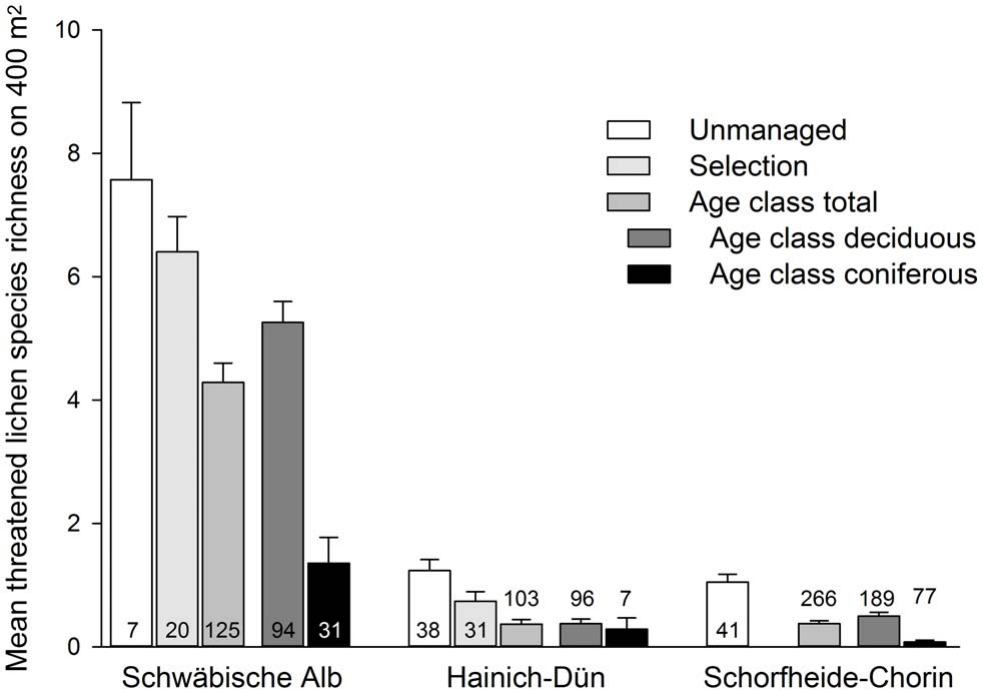
Mean species richness of threatened lichens (+SE) per plot for each of the forest management types in the three study regions.

Species richness per plot of all lichens and of threatened lichens was significantly higher in the Schwäbische Alb than in both other regions (Figures 1, 2). Total species richness per plot was on average 18.6 (SD 8.6) in the Schwäbische Alb, 5.0 (2.7) in Hainich-Dün, and 6.0 (3.0) in Schorfheide-Chorin.

### Management effects on lichen species richness

Total and corticolous lichen species richness did not differ between unmanaged and age-class forests. However, unmanaged forests harbored 21.9% more threatened lichen species than managed age-class forests (Tables 2, 5).

**Table 5:** Mean lichen species richness per 400 m^2^ (untransformed mean ±SE) in unmanaged and age-class forests, and in deciduous and coniferous age-class forests, in total and for the three study regions.

	*N*	All lichens		Threatened lichens		Corticolous lichens		Saxicolous lichens		Lignicolous lichens	
*Total*											
Unmanaged	86	7.3	(0.6)	1.7	(0.2)	5.4	(0.4)	1.3	(0.3)	0.6	(0.1)
Age class_total_	494	8.7	(0.3)	1.4	(0.1)	6.9	(0.3)	0.5	(0.1)	1.2	(0.1)
Age class_deciduous_	379	9.1	(0.4)	1.6	(0.1)	7.3	(0.3)	0.5	(0.1)	0.9	(0.1)
Age class_coniferous_	115	7.4	(0.5)	0.4	(0.1)	5.4	(0.4)	0.2	(0.1)	2.0	(0.2)
*Schwäbische Alb*											
Unmanaged	7	23.1	(3.2)	7.6	(1.3)	14.0	(2.6)	6.9	(2.2)	0.7	(0.4)
Age class_total_	125	18.2	(0.8)	4.3	(0.3)	13.8	(0.7)	1.2	(0.2)	1.2	(0.2)
Age class_deciduous_	94	20.1	(0.9)	5.3	(0.3)	15.4	(0.7)	1.3	(0.2)	1.0	(0.2)
Age class_coniferous_	31	12.5	(1.4)	1.4	(0.4)	8.7	(1.2)	0.7	(0.2)	1.8	(0.4)
*Hainich-Dün*											
Unmanaged	38	5.1	(0.3)	1.2	(0.2)	4.7	(0.3)	0.1	(0.1)	0.3	(0.1)
Age class_total_	103	4.6	(0.3)	0.4	(0.1)	3.8	(0.2)	0.4	(0.1)	0.3	(0.1)
Age class_deciduous_	96	4.6	(0.3)	0.4	(0.1)	3.8	(0.2)	0.4	(0.1)	0.3	(0.1)
Age class_coniferous_	7	4.9	(0.9)	0.3	(0.2)	4.0	(1.0)	0.0	(0.0)	0.4	(0.4)
*Schorfheide-Chorin*											
Unmanaged	41	6.6	(0.5)	1.0	(0.1)	4.5	(0.3)	1.6	(0.2)	0.9	(0.2)
Age class_total_	266	5.9	(0.2)	0.4	(0.0)	4.8	(0.2)	0.2	(0.0)	1.5	(0.1)
Age class_deciduous_	189	6.0	(0.2)	0.5	(0.1)	5.1	(0.2)	0.2	(0.0)	1.2	(0.1)
Age class_coniferous_	77	5.6	(0.3)	0.1	(0.0)	4.2	(0.2)	0.0	(0.0)	2.2	(0.2)

In general, total and corticolous lichen species richness did not differ among management types of deciduous stands. However, among those stands the species richness of threatened lichens was highest in unmanaged, intermediate in selection and lowest in age-class forests (Tables 3, 6). In general, threatened lichen species richness was higher in deciduous than in coniferous age-class forests. This was also the case for corticolous lichen species richness in the Schwäbische Alb and Schorfheide-Chorin, and for the total lichen species richness only in the Schwäbische Alb. In contrast, the species richness of lignicolous lichens was generally higher in coniferous than in deciduous age-class forests (Tables 4, 5; Figures 1, 2). Thus, promoting stands with site-typical composition of tree species appears important for promoting lichen species richness and threatened lichen species in forests.

**Table 6:** Mean lichen species richness per 400 m^2^ (untransformed mean ±SE) in unmanaged and differently managed deciduous forests in total, and for the Schwäbische Alb and Hainich-Dün regions.

	*N*	All lichens		Threatened lichens		Corticolous lichens		Saxicolous lichens		Lignicolous lichens	
*Total*											
Unmanaged	45	7.9	(1.1)	2.2	(0.4)	6.2	(0.7)	1.2	(0.5)	0.4	(0.1)
Age class_deciduous_	190	12.3	(0.7)	2.8	(0.2)	9.5	(0.6)	0.8	(0.1)	0.7	(0.1)
Selection_deciduous_	51	11.2	(1.1)	3.0	(0.5)	8.1	(0.8)	1.7	(0.3)	0.5	(0.1)
*Schwäbische Alb*											
Unmanaged	7	23.1	(3.2)	7.6	(1.3)	14.0	(2.6)	6.9	(2.2)	0.7	(0.4)
Age class_deciduous_	94	20.1	(0.9)	5.3	(0.3)	15.4	(0.7)	1.3	(0.2)	1.0	(0.2)
Selection_deciduous_	20	19.1	(1.3)	6.4	(0.6)	12.7	(1.1)	4.0	(0.5)	0.7	(0.2)
*Hainich-Dün*											
Unmanaged	38	5.1	(0.3)	1.2	(0.2)	4.7	(0.3)	0.1	(0.1)	0.3	(0.1)
Age class_deciduous_	96	4.6	(0.3)	0.4	(0.1)	3.8	(0.2)	0.4	(0.1)	0.3	(0.1)
Selection_deciduous_	31	6.1	(0.6)	0.7	(0.2)	5.1	(0.5)	0.2	(0.1)	0.4	(0.1)

In the Schwäbische Alb, species richness of saxicolous lichens was 428% higher in unmanaged than in deciduous age-class forests and also 73% higher than in selection forests (Tables 3, 6).

### Standing biomass and lichen species richness

Overall, old forests with large quantities of standing biomass were slightly richer in corticolous lichen species than were young forests with low quantities of standing biomass: we found an increase of 1.2 species with an increase of 500m^3^ standing biomass per ha (Table 2). Among deciduous stands this relationship was even more pronounced (+2.0 species/500 m^3^). Furthermore, threatened lichen species richness increased with standing biomass, more strongly for the Schwäbische Alb (+2.6 species/500 m^3^) than for the Hainich-Dün (+0.6 species/500 m^3^), as indicated by the significant region - by - standing biomass interaction (Table 3). Overall, we found opposing effects of standing biomass on the richness of corticolous and threatened lichen species between deciduous and coniferous age-class forests (significant standing biomass - by - management interaction; Table 4). High standing biomass was associated with higher richness of corticolous (+2.0 species/500 m^3^) and of threatened lichen species (+0.7 species/500 m^3^) in deciduous stands but with lower species richness of corticolous (-1.3 species/500 m^3^) and of threatened lichen species (-1.2 species/500 m^3^) in coniferous stands (Table 4). These findings indicate that the conservation of old forests dominated by native broadleaved tree species might enhance species richness of corticolous lichens and promote suitable habitats for threatened lichen species. Interestingly, the richness of lignicolous lichen species decreased (-0.7 species/500 m^3^) with an increase in standing biomass (Tables 2, 3, 4). This was probably because of higher amounts of deadwood in younger stands due to recent timber harvesting.

### Cover by deadwood and rocks and lichen species richness

Overall, deadwood cover decreased with standing biomass (*N*  =  631, *r*  =  -0.1762; *p* < 0.0001). Lignicolous lichen species richness was generally positively related to the cover of deadwood, increasing by 0.7 species per 10% increased deadwood cover (Tables 2, 3, 4). These findings indicate that increasing the amount of deadwood in forests may lead to an increase of lichen species richness.

Overall, species richness of saxicolous lichens increased by 1.2 species per 1% increased rock cover (Tables 2, 3, 4). However, effects varied among regions and management types due to varying cover values (Table 1).

## Discussion

### Differences in lichen species richness among the regions

Our results showed strong differences in lichen species richness between the Schwäbische Alb and the two other regions, although all forests have similar management methods. These differences are not related to the protection status of the regions or to management activities but rather to the former intensity of atmospheric pollutants, especially SO_2_, which was responsible for the decline of many lichen species in Germany [1]. Until the strong decrease of SO_2_ pollution around 1990 in Germany, SO_2_ deposition was low in the Schwäbische Alb, high in Schorfheide-Chorin and very high in Hainich-Dün ([25]; Table 1). Regionally, this resulted in a shift in lichen species composition to “lichen deserts” with only a few toxitolerant species [7], [8]. Today, levels of SO_2_ pollution have considerably decreased and many species are re-colonizing these areas [7], [9]. However, the characteristic lichen communities have not yet recovered completely and therefore current lichen community composition in these areas might still be influenced by former air pollution. Furthermore, the higher mean rock cover and mean annual precipitation in the Schwäbische Alb than in the other regions (Table 1), might also contribute to the much higher lichen species richness in the Schwäbische Alb.

### Management effects on lichen species richness

In our study, total and corticolous lichen species richness did not differ between unmanaged and age-class forest sites. Bergamini et al. [15] and Paillet et al. [13], who used much smaller datasets from other European countries, found higher total and corticolous lichen species richness in unmanaged compared with managed forests. Our results also contrast with those of Friedel et al. [18], who found higher species richness of corticolous lichens in 45 unmanaged than in 45 managed European beech forests in northeastern Germany, and with the findings of Rudolphi and Gustafsson [29], who compared 19 unmanaged and 19 young stands, originating from clear-cutting, in boreal forests in Sweden. The differences among the studies might be because the investigated unmanaged stands that we surveyed had not yet reached the degeneration phase and still showed signs of former management, such as a fairly even-aged structure and a dense canopy cover (as outlined in [30]). However, overall, unmanaged forests harbored more threatened lichen species than managed age-class forests. This confirms the particular importance of unmanaged forests for the conservation of lichen species with long generation times [3] or with specific habitat requirements that mean they are restricted to old growth forests [21]. Other management-related disturbances such as logging trails had no negative effects on lichen species richness, showing that management involving forestry equipment does not need to reduce the species richness of lichens if suitable habitats are spared.

We found no differences in total and corticolous lichen species richness among deciduous forests. However, we did find highest species richness of threatened lichens in unmanaged forests, with intermediate richness in selection forests, and the lowest richness in age-class forests. This result underlines the importance of silvicultural methods which ensure temporal forest continuity for many lichen species. This is line with several other studies pointing out the importance of stand continuity to preserve lichen species richness and communities with rare or threatened lichen species [13], [22], [31]–[34]. Alternatively, patches of old trees could be retained in forests to conserve lichen species richness in managed stands. Peterson and McCune [35] compared 51 forest stands in Oregon and found that retention patches or old trees were essential for the persistence of lichen communities that depend on old-growth forest conditions.

Our study provides further evidence for the idea that older forests harbor more lichen species and particularly more threatened species [5], [36]–[39]. These positive effects might probably be explained by characteristics of old trees which make them better lichen habitats. These include, a larger bark surface which increases the probability of colonization [40], pronounced bark textures including crevices [22], [40] and rot holes [41], which provide a range of microhabitats, and the fact that many lichen species associated with old-growth conditions can only establish on older trees [42]. The retention of numerous mature to over-mature trees in forests appears important for maintaining a high species richness of epiphytic lichens [43]–[45] and to conserve rare and threatened lichen species in managed forests [21], [29], [46], [47]. These trees can act as population centers for the dispersal of lichen propagules and provide refuges for those species which depend on old trees [3], [48]–[50].

Interestingly, species richness of lignicolous lichens decreased with higher standing biomass. Most likely, this finding was due to the higher amounts of deadwood, following recent timber harvesting, in our younger stands of low standing biomass than in our older stands of high standing biomass. Thus, we suggest that increasing the amount of deadwood in older forests may lead to an increase in lichen species richness, in agreement with the findings of Moning et al. [19] based on 113 plots within the National Park Bavarian Forest (Germany).

Clear cutting generally replaced site-characteristic forests with even-aged and homogeneous plantations often with different tree species and this had a very pronounced effect on lichen species richness and composition [3], [44], [51]. In general, we found more threatened lichen species in deciduous forests than in coniferous forests. In the Schwäbische Alb we also found more corticolous lichen species in deciduous forests. This is in line with the findings of Humphrey et al. [36] who observed higher lichen species richness in native deciduous stands than in conifer plantations in different forest sites in Britain. Furthermore, Neitlich and McCune [52] pointed out the importance of deciduous tree patches interspersed in young coniferous plantations for both lichen species richness and the species richness of specialized lichens with high conservation value. Thus, promoting stands or retaining patches with a site-typical composition of tree species appears important for promoting lichen species richness and threatened lichen species in forests.

### Rock cover and lichen species richness

Overall we found positive relations between rock cover and the species richness of saxicolous lichens. Clearly, not just the presence but also the quantity of this substrate matters for lichen species richness. As managed stands occur on more easily accessible, flat sites where rocks are scarce, the higher species richness of saxicolous lichens in unmanaged than in age-class or selection forests might well be related to the lower rock quantity in unmanaged forests, rather than to the absence of management per se.

### Deadwood cover and lichen species richness

In our study, the species richness of lignicolous lichens generally increased with increasing cover of deadwood. Similarly, in their deadwood-focused study Caruso et al. [53] reported a positive relationship between deadwood volume and the species richness of lignicolous lichens in 30 stands of planted boreo-nemoral Swedish forests. Thus, similar to Moning et al. [19] and Humphrey et al. [36] we recommend actively enhancing deadwood quantity, diversity of types and decay stages of standing deadwood and deadwood on the ground.

### Lichen species richness in tree crowns

We probably have underestimated the overall lichen richness because we could not assess species restricted to tree crowns, as this would have required lengthy tree climbing of thousands of trees. According to this difficulty, we are not aware of any other study that attempted to assess lichen diversity in tree crowns of differently managed forests.

## Conclusions

Our findings demonstrate the importance of management systems that ensure stand continuity for lichen conservation. Clearly, the conservation of old forests with high standing biomass is absolutely necessary to maintain a high species richness of lichens and to promote threatened lichen species. To increase total and threatened lichen species richness without overly compromising timber production, our results, combined with those of the other studies discussed above, lead us to the following recommendations for managed forests: (1) to promote silvicultural methods that assure stand continuity, e.g. by selection cutting rather than clear cutting and shelterwood logging, (2) to conserve retention patches with groups of old, mature to over-mature trees in managed forests, (3) to conserve at least single mature to over-mature trees which may serve as sources for colonization, (4) to select, as far as visibly obvious, such retention trees of high lichen abundance and species richness composition and population size rather than selecting them at random, (5) to promote stands of native deciduous tree species instead of coniferous plantations, and (6) to increase the amount of deadwood in forests.
